# Circulant Singular Spectrum Analysis and Discrete Wavelet Transform for Automated Removal of EOG Artifacts from EEG Signals

**DOI:** 10.3390/s23031235

**Published:** 2023-01-21

**Authors:** Jammisetty Yedukondalu, Lakhan Dev Sharma

**Affiliations:** School of Electronics Engineering, VIT-AP University, Amaravati 522237, Andhra Pradesh, India

**Keywords:** electroencephalogram (EEG), electrooculogram (EOG), circulant singular spectrum analysis (CiSSA), discrete wavelet transform (DWT), kurtosis, energy

## Abstract

**Background:** Portable electroencephalogram (EEG) systems are often used in health care applications to record brain signals because their ease of use. An electrooculogram (EOG) is a common, low frequency, high amplitude artifact of the eye blink signal that might confuse disease diagnosis. As a result, artifact removal approaches in single EEG portable devices are in high demand. **Materials:** Dataset 2a from the BCI Competition IV was employed. It contains the EEG data from nine subjects. To determine the EOG effect, each session starts with 5 min of EEG data. This recording lasted for two minutes with the eyes open, one minute with the eyes closed, and one minute with eye movements. **Methodology:** This article presents the automated removal of EOG artifacts from EEG signals. Circulant Singular Spectrum Analysis (CiSSA) was used to decompose the EOG contaminated EEG signals into intrinsic mode functions (IMFs). Next, we identified the artifact signal components using kurtosis and energy values and removed them using 4-level discrete wavelet transform (DWT). **Results:** The proposed approach was evaluated on synthetic and real EEG data and found to be effective in eliminating EOG artifacts while maintaining low frequency EEG information. CiSSA-DWT achieved the best signal to artifact ratio (SAR), mean absolute error (MAE), relative root mean square error (RRMSE), and correlation coefficient (CC) of 1.4525, 0.0801, 18.274, and 0.9883, respectively. **Comparison:** The developed technique outperforms existing artifact suppression techniques according to performance measures. **Conclusions:** This advancement is important for brain science and can contribute as an initial pre-processing step for research related to EEG signals.

## 1. Introduction

### 1.1. Motivation

The study of brain illnesses, motor functions, cognitive loads, attention spans, and levels of attention are all greatly aided by the electroencephalogram (EEG), which records the electrical activity of the brain [1,2,3,4,5]. EEG signals are often employed in applications being the brain–computer interface (BCI) system, which extracts the EEG signal information and forwards it to a physical system as a command signal. Several approaches for extracting EEG components for band identification in BCI applications have been presented [6,7]. Biological artifacts, namely electrooculogram (EOG), electrocardiogram (ECG), and electromyogram (EMG), usually contaminate recorded EEG data. The EOG artifact is caused by eye blinking or movement and contaminates the EEG signal. In this work, we referred to the EOG artifact as an eye blink artifact since it is an unregulated, instinctive behavior that occurs every five seconds. The EEG low frequency spectrum (0.5–12 Hz) is generally contaminated by the eye blink artifact [8]. Because of this, the informational value of the EEG data is affected and its removal is important. Therefore, removing EOG artifacts is a vital task to be performed before analyzing the EEG data [9].

### 1.2. Related Work

In Mannan et al. [10,11,12], various single and multi-channel approaches were established. By combining overlap segmented adaptive singular spectrum analysis (Ov-ASSA) and adaptive noise canceler (ANC), Noorbasha et al. [13] established a single channel artifact elimination system with clear improvements in epilepsy identification. A unique approach, designated as singular spectrum analysis, independent component analysis, and stationary wavelet transform (SSA-ICA-SWT) [14], was introduced according to the combination of SSA and ICA with a SWT, and it produced the good artifact elimination work different to current methods such as SSA, SSA-ANC, and SSA-ICA. Researchers have been particularly interested in blind source separation (BSS), which does not require any prior knowledge of EOG and is useful for removing artifacts from multichannel contaminated EEG data [15]. To eliminate artifacts from multichannel EEG recordings, BSS approaches such as canonical correlation analysis (CCA), ICA were applied [16,17,18,19]. In comparison to the CCA approach, the ICA methodology has been widely used to eliminate eye blink artifacts from EEG recordings [17,19]. For successful removal of eye blink artifacts from multi- channel EEG data, numerous various methods were used with ICA [20,21,22]. The drawback of ICA is that there needs to be a very large number of channels employed to collect the data relative to the underlying artifactual sources. To suppress the artifact from EEG data, the artifact subspace reconstruction (ASR) approach was developed in Chang et al. [23]. The effectiveness of the approach is controlled by the parameter. Despite doing a comprehensive analysis to identify the cut-off value, choosing it incorrectly might cause the EEG data to be lost [23,24].

The need for at-home healthcare observation has lately grown as a result of an increase in chronic illnesses as well as population aging [25]. Portable EEG devices have been used in several studies for a variety of purposes, including mental state investigation in stroke sufferers, driver fatigue, sleep disorders, and event related potential (ERP)-based BCI applications [26,27]. Traditional ERP analysis treats ERPs as being time-locked to the commencement of a motor or sensory stimulus. ERPs are distinct variations in the brain’s electrical potential. The examination of ERP signals is useful for memory, language, and attention research in psychology. An ERP is obscured in single trials by background EEG activity and artifact contamination of the recordings. Hence, a common ERP analysis calls for averaging the data over several trials to reveal the ERP signal of interest [28]. The use of single-trial ERP analysis is growing, and a variety of techniques are being used to denoize ERPs in order to eliminate artifacts and underline EEG activity [29]. Denoizing techniques include Wiener filtering and autoregressive modeling. Because wavelets more effectively manage the nonstationary and nonlinear characteristics of ERPs, they are becoming more widely used for this application. Wavelets offer a helpful time-frequency decomposition for ERP research, but they are unable to remove undesired EEG activity. In these circumstances, wavelet denoizing could add false ERP waves [29]. Wavelets cannot be used before artifact-contaminated trials have been eliminated, which means that valuable EEG samples are lost. The main barrier to such denoizing is ocular artifacts, which appear in the same low frequency regions as the ERP signals and skew the ERP activity [30].

To reduce cognitive load on the patient, portable EEG devices by fewer EEG channels, particularly single EEG channel devices, have recently been designed [31,32]. As a result, multichannel ICA and ASR approaches cannot be employed to suppress eye blink artifacts in EEG recordings by a single channel due to collecting the data relative to the underlying sources. As a result, new approaches for processing EEG data that tailored are required. One way of processing EEG data by single channel is to apply an adaptive filter (AF). He et al. [33] were the first to discuss the benefit of AF to eliminate eye blink artifacts from EEG data but it needs reference signals to eliminate artifacts from EEG single channel data. In Peng et al. [34], AF and DWT are combined to address this issue. In this method, the contaminated EEG signal is employed to produce the reference signal for AF-DWT. The AF then removes the eye blink artifact from the EEG data using the predicted eye blink artifact signal as a reference signal. The reference signal for the AF is computed using DWT with a contaminated EEG signal in this technique. Hence the adaptive filter uses the predicted eye blink artifact signal as a reference signal to eliminate the eye- blink artifact from the EEG signal. The Savitzky–Golay (SG) filter has recently been used to predict the reference signal required by an AF [35]. To reduce eye blink artifacts from single channel EEG, the variational mode extraction (VME) and DWT approaches were recently coupled [36]. The EOG artifact period is initially determined using VME in this manner. The EOG of the EEG signal is then filtered using the DWT method. Although the non-artifact segments of the EEG signal were unaffected by this approach, the EOG component is removed in part from the contaminated EEG signal. An ensemble empirical mode decomposition (EEMD) using adaptive noise, which uses data-driven decomposition to suppress eye blink artifacts from EEG signal, is also described [37,38,39]. The EEG signal’s non-artifact portions are altered by this technique.

A subspace-based technology called SSA is employed to extract low frequency, oscillatory, and noisy elements from single time series data [40,41]. This approach has been used recently to handle biological signals [42,43,44]. It was first addressed in [45] whether SSA might be conformed to eliminate eye blink artifacts from EEG data of single channel. In conventional SSA, however, determining the required signal eigenvectors is an important phase. As a result, in [46], new criteria for identifying the eigenvectors that are used to reconstruct the required signal were presented. SSA was used amid an AF in [46] to improve the AF performance over the technique in [34]. Additionally, single channel EEG recordings have been processed to ICA using SSA [47]. SSA was newly used as a smoothing filter to reduce the EOG phenomenon from an EEG signal [48].

The circulant singular spectrum analysis (CiSSA), which is based on circulant matrixes, can be used to analyze each and every signal in a time series, and it inherently ties the frequency of interest with the specific principal components (PCs) in [49]. Because their eigenstructure has been defined as a function of frequency, their eigenvectors and eigenvalues may be naturally associated with any specific frequency. The circulant matrixes have proven to be acceptable in this specific setup. As part of the overall setup, CiSSA also uses circulant matrixes to select the eigenvectors and eigenvalues related to each frequency [50]. The CiSSA was used to decompose the EOG artifacts linked EEG signal into intrinsic mode functions (IMF’s) are related to low frequency artifacts. By comparing the threshold value with the kurtosis and energy, the initial IMFs can be separated from the remainder, and the remaining IMFs may be combined together to apply inverse CiSSA (ICiSSA). Next, we used a four-level DWT of mother wavelet db4 to decompose the EOG-related IMFs into approximate and detailed coefficients, to make zero approximate coefficients due to low frequency EOG-related components, and applied an inverse DWT (IDWT) to reconstruct the signal. The resultant signal is an EOG free EEG signal and also obtain excellent correlation coefficient (CC), relative root mean square error (RRMSE), mean absolute error (MAE), and signal to artifact ratio (SAR). On synthetic and real single channel EEG data, the work of the proposed framework (CiSSA-DWT) is examined. The findings show that it outperforms existing methods and current data- driven decomposition methods.

The rest of the article is structured in this manner: The proposed framework is detailed in Section 2. Performance metrics are specified in Section 3. Simulation findings are described in Section 4, discussed in Section 5, and the conclusions are presented in Section 6.

## 2. Materials and Methods

### 2.1. Materials

**Figure 1:**
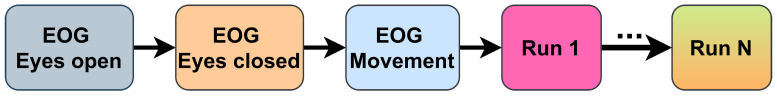
Experimental protocol for EEG recording.

In this work, BCI Competition IV Data set 2a was used. It comprises electroencephalographic (EEG) data from nine subjects. Four different motor imagery tasks were included in the cue-based BCI paradigm: the left hand (class 1), right hand (class 2), both feet (class 3), and tongue (class 4). For each subject, two sessions on various days were recorded. There are six runs in each session, separated by brief rest periods. 48 trials (12 for each of the 4 possible classes) make up one run, for a total of 288 trials each session. Approximately 5 min of EEG data are collected at the start of each session to estimate the EOG influence. This recording was divided into three blocks: 2 min with eyes open, 1 min with eyes closed, and 1 minute with eye movements as shown in Figure 1. All signals were recorded monopolarly, with the left mastoid serving as reference and the right mastoid as ground. In addition to the 22 EEG channels, three mono-polar EOG channels were recorded [51].

#### Signal Acquisition

The EEG signals were sampled at 250 Hz and band pass filtered (with the 50-Hz notch filter enabled) between 0.5 and 100 Hz. The sensitivity of the amplifier was set to 1 mV. The EOG channels are provided for the subsequent application of artifact processing methods and must not be used for classification.

### 2.2. Methods

**Figure 2:**
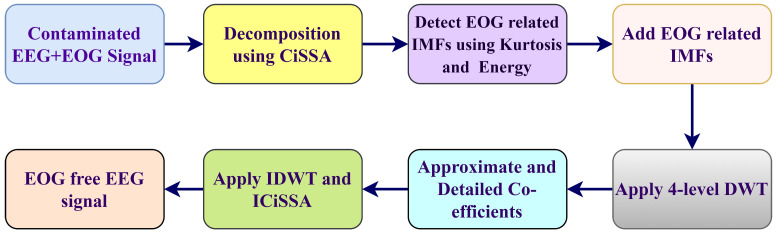
Proposed methodology diagram.

Figure 2 depicts the proposed framework. In more detail, the proposed method is divided into four phases. In the first phase, CiSSA was applied to decompose EEG signal into IMFs [52,53]. The IMFs relevant to EOG would be automatically selected in the second phase by analyzing kurtosis and energy. The DWT method was used to decompose EOG-related IMFs in the third phase. To identify and eliminate EOG-linked IMFs, the fraction of low frequency band power of IMFs, CC value among every IMF and the EOG component were employed in the fourth phase. Finally, using the inverse transform of CiSSA and DWT, reconstruction obtained the EEG signal free of EOG artifacts.

### 2.3. Decomposition of EEG Signals with CiSSA

In refs. [49,54], they introduced the CiSSA approach, which is a non-parametric signal extraction method. The four steps of CiSSA are grouping, diagonal averaging, decomposition, and embedding. CiSSA feasible executed in the four steps below:

**Step-1:**  **Embedding** In this step to construct a R×S trajectory matrix *A*, S=t−R+1, from the original time series as described in the following:
  (1) $$A=\left(a_{1}\left|...\right|a_{N}\right)=\left(\begin{array}{ccccc} a_{1} & a_{2} & a_{3} & \cdots & a_{S}\\ a_{2} & a_{3} & a_{4} & \cdots & a_{S+1}\\ \vdots & \vdots & \vdots & \cdots & \vdots \\ a_{R} & a_{R+1} & a_{R+2} & \cdots & a_{t} \end{array}\right)$$
  where $a_{i}={\left(a_{i},....a_{i+R+1}\right)}^{\prime }$ specifies the vector R×1 with origin at time.
**Step-2:**  **Decomposition** Construct the circulant matrix $C_{M}$, which has the following components:
  (2) $${\hat{M}}_{n}=\frac{R-n}{R}{\hat{n}}_{n}+\frac{n}{R}{\hat{n}}_{R-n},n=0,...R-1$$
  calculate the eigenvalues of ${\hat{\mu }}_{b}$ of $C_{M}$ as follows:
  (3) $$\mu_{R,b}=f\left(\frac{b-1}{R}\right)$$
  Collaborate the *k*th eigenvalue and the corresponding eigenvector with frequency $V_{b}=\frac{b-1}{R},b=1,..R$.
**Step-3:**  **Grouping** We obtain ${\hat{\mu }}_{b}=\mu_{R}+2-b$ when we specify the analogy of power spectral density (PSD). Then, because their associated eigenvectors are complex, these are complex conjugate pairs, $j_{b}=j_{R+2-b}^{*}$, where j* denotes the complex conjugate of *j*. To calculate related elements, we turn them into pairs of real eigenvectors. To make the fundamental matrixes, we’ll need to make two-element groups $A_{B}=b,R+2-b$ for b=2,...N with A1= 1 and $A_{\frac{R}{2}}+1=\frac{R}{2}+1$ if *R* is even. Then, using the frequency $D_{A_{b}}$, compute the elementary matrix as the total of two elementary matrixes $D_{b}$ and $D_{R+2b}$, those are connected to the eigenvalues ${\hat{\mu }}_{b}$ and ${\hat{\mu }}_{R+2-b}$ and the frequency $V_{b}=\frac{b-1}{R}$,
  (4) $$\begin{array}{c} D_{A_{b}}=D_{b}+D_{R+2-b}\\ D_{A_{b}}=\left(j_{b}j_{b}^{H}D+j_{R+2-b}j_{R+2-b}^{H}\right)D\\ D_{A_{b}}=2\left(Y_{j_{b}}Y_{j_{b}}^{{}^{\prime }}+Z_{j_{b}}Z_{j_{b}}^{{}^{\prime }}\right)D\\ D_{A_{b}}=\left(j_{b}j_{b}^{H}+j_{b}^{*}j_{b}^{{}^{\prime }}\right)D \end{array}$$
  where $Y_{j_{b}}$ is real part of $j_{b}$, imaginary part is $Z_{j_{b}}$,and $j^{H}$ denotes the conjugate transpose of *j*.
  (5) $$j_{b}=R^{-1/2}\left(j_{b,1}...j_{b,R}\right)$$
**Step-4:**  **Reconstruction** Each matrix $D_{Oi}$ is averaged diagonally to produce the newest time series of length t equal to the preceding one. It is equivalent to calculating the mean of the non-diagonal elements of $D_{Oi}$, or hankelizing this matrix, which is denoted by the operator H (.). The Vautard-Ghil replacement, also known as the Toeplitz SSA, conducts orthogonal diagonalization from a different matrix $G_{t}=\left(G_{oi}\right)$ under the premise that it is stationary and has a mean value of a non-zero average.
  (6) $$G_{oi}=\frac{1}{t-|o-i|}\sum_{n=1}^{t-|o-i|}D_{n}D_{n+|o-i|}$$

**Figure 3:**
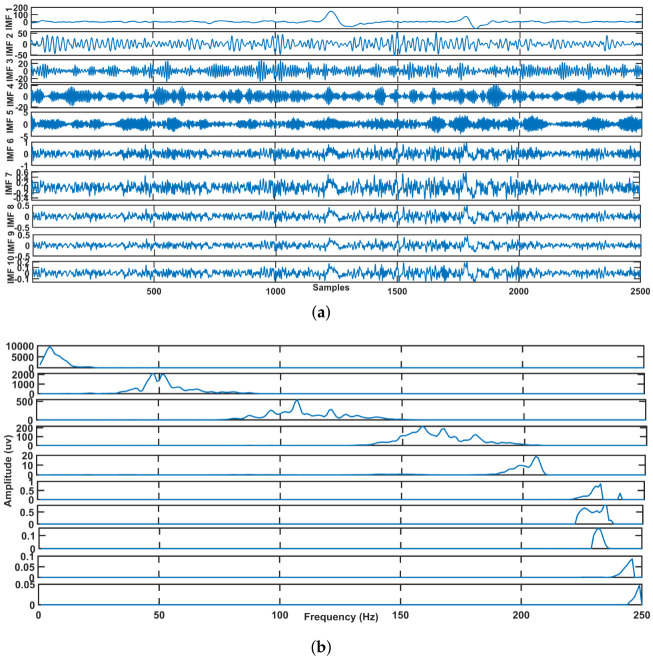
10-level decomposition of EEG signal IMFs and PSD. (**a**) Decomposition of contaminated EEG signal using CiSSA. (**b**) Power Spectral Density.

As the original series sample lagged variance covariance matrix, the $G_{t}$ is a symmetric Toeplitz matrix. The $b^{th}$ eigentriple is named after the set ($t_{b},V_{b},j_{b}$). Figure 3 depicts the decomposed signal (IMF’s) and PSD of IMFs by CiSSA. As a result, the resulting signal window length is $W_{d}$, which is defined as Equation (7)

(7) $$W_{d}=f_{d}\times f_{s}$$

Frame duration is $f_{d}$, while sampling frequency is $f_{s}$.

### 2.4. EOG Related IMFs Selection

After the IMFs have been obtained, this phase aims to identify EOG-related IMFs automatically for further processing. We employed kurtosis and energy to find EOG-related components in this study.

#### 2.4.1. Kurtosis

The cusp of the peak is naturally represented by the statistical quantity known as kurtosis, which measures how steep a sample distribution. In comparison to other IMFs, the kurtosis values of those associated with EOG are much higher [18]. The following equation may be used to compute the kurtosis of the *i*th IMF:

(8) $$K_{i}=m_{4}-m_{2}^{2}$$

Here $m_{n}$ denotes that data’s *n*th central moment and is defined as follows:

(9) $$m_{n}=E{\left(x-m_{1}\right)}^{n}$$

Here mean value is $m_{1}$, and the expectation function is E().

#### 2.4.2. Energy

The energy $E_{s}$ of a continuous time signal is the region under the squared magnitude of the estimated signal s(t). The following equation may be used to calculate the energy of the IMFs:

(10) $$E_{s}=\left(s\left(t\right),s\left(t\right)\right)=\int_{-\infty }^{\infty }{\left|s\left(t\right)\right|}^{2}dt$$

When criteria kurtosis and energy thresholds $K_{t}$ and $E_{t}$ were established, respectively, the IMFs whose kurtosis and energy exceeded the pertinent thresholds were classified as being associated with the EOG.

$$EZ={\left[ez_{1}\left(t\right),ez_{2}\left(t\right),...ez_{n}\left(t\right)\right]}^{T}\in S^{n\times M}$$

where *n* represents how many EOG components there are in total. DWT would then receive them to continue the decomposition process.

### 2.5. EOG Component Decomposition Using DWT

**Figure 4:**
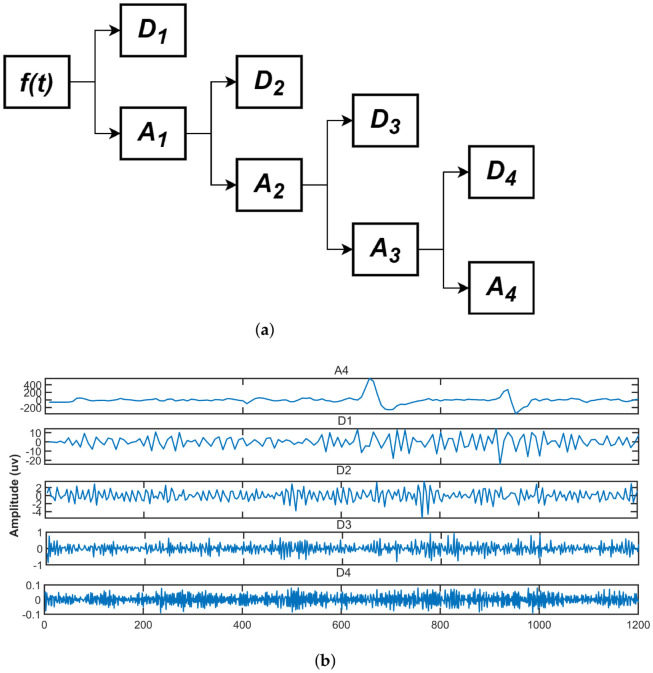
4-level tree and decomposition of EOG using DWT. (**a**) 4-level DWT of IMF1 signal decomposed by CiSSA. (**b**) Approximate and detailed coefficients of IMF1 using 4-level DWT.

The wavelet transform is a Fourier transform-based time-frequency analysis tool. The wavelet coefficients can represent signal information in both the time and frequency domains. As a result, the wavelet transform is frequently employed in the analysis of biological signals, particularly non-stationary signals such as EEG. The DWT has a very quick computation speed, making it ideal for real-time artifact reduction in EEG. Furthermore, because most real signals must be handled discretely after sampling, the discrete wavelet transform is commonly used. The following is the DWT:

(11) $$wt_{f}\left(m,n\right)=<f\left(t\right),\phi_{m,n}\left(t\right)>=\int_{-\infty }^{\infty }f\left(t\right){\bar{\phi }}_{m,n}\left(t\right)dt,\;m,n\in X$$

where $\phi_{m,n}\left(t\right)$ is the mother wavelet function’s binary expansion and shift, and ϕ(t), *n*, and *m* are the frequency resolution and time shifts, respectively. The conjugate of $\phi_{m,n}\left(t\right)$ is ${\bar{\phi }}_{m,n}\left(t\right)$. The Mallat technique is used to create a 4-level decomposition of signal f(t), and the wavelet coefficients are as follows:

(12) $$a_{m,n}=\sum_{k\in Z}a_{m-1,k}h_{0}\left(k-2n\right),$$

(13) $$d_{m,n}=\sum_{k\in Z}a_{m-1,k}h_{1}\left(k-2n\right)$$

where $a_{m,n},d_{m,n}$ are approximate and detailed coefficients of *m*th scale (m=1,2,...,L). $h_{0},h_{1}$ are low and high frequency filters, respectively, and determined by selected wavelet basis. Figure 4 depicts a typical four-level decomposition tree. Where $A_{m}$ denotes the approximation vector and $D_{m}$ denotes the *m*th scale’s detail one. Furthermore, the decomposition is consistent with the following equation:

(14) $$f\left(t\right)=A_{4}+\sum_{m=1}^{4}D_{m}$$

The approximate coefficients are low frequency, which is an EOG artifact as shown in Figure 4, so we make them the zero of the approximate coefficients.

### 2.6. EEG Signal Reconstruction without EOG

In the previous stage, the approximate coefficients that reflect EOG artifacts were eliminated. The signal was rebuilt using the inverse transformation of DWT of detailed coefficients. The clean EEG signals might eventually be rebuilt by applying ICiSSA to EOG less components.

#### Inverse DWT and CiSSA

IDWT simply involves applying DWT in reverse. By adding zeros between each coefficient and increasing their length, the DWT coefficients are first upsampled (the approximation and the detail coefficients are treated separately). The IDWT is defined as follows:

(15) $$f\left(t\right)=\sum_{m=-\infty }^{\infty }\sum_{n=-\infty }^{\infty }wt_{f}\left(m,n\right)\phi_{m,n}\left(t\right),\;m,n\in X$$

Inverse CiSSA is the result of using CiSSA reverse, as seen in Equation (6). The EOG free EEG signals were reconstructed by ICiSSA and IDWT using Equations (15) and (6).

## 3. Performance Metrics

We have specified several metrics to compare the proposed technique to current methods in this section. However, as for this study, we used the EEG mixing model described below. The ground truth EEG and the EOG artifact are represented by the vectors *q* and *k*, respectively. The contaminated EEG signal (s), then is defined as

(16) s=q+pk

The presence of EOG artifacts in the raw EEG signal is represented by the constant *p* (Constant for artifact mixing). The SNR of contaminated EEG is low when p>1 (EOG artifact is more prominent) and high when p<1. The effectiveness of the presented artifact elimination technique is examined using real and synthetic EEG data. We defined the following two metrics to evaluate the outcome of the suggested artifact removal approach with that of current methods on synthetic EEG signals: CC and RRMSE. However, two metrics, namely SAR and MAE, are used to evaluate the real EEG signals. Through the use of these measurements, it is possible to objectively assess if a method works without changing the EEG signal.

### 3.1. RRMSE

The RRMSE can be calculated by taking the ground truth or real EOG artifact as *k* and the estimated EOG artifact derived by the artifact removal process as $\hat{k}$:

(17) $$\mathrm{RRMSE}=\sqrt{\frac{\sum_{n=1}^{N}{\left[k\left(n\right)-\hat{k}\left(n\right)\right]}^{2}}{\sum_{n=1}^{N}k^{2}N}}\times 100\%$$

where *N* is the signal sample number. The difference between *k* and $\hat{k}$ (Numerator of Equation (17)) will be modest when an artifact removal technique properly calculates the EOG artifact from the EEG signal, and Consequently, for an effective artifact removal approach, the RRMSE value will be low.

### 3.2. CC

The correlation between two signals is represented by CC, a statistically-based performance metric. The difference between the estimated EOG $\hat{k}$ component and the ground truth EOG *k* component is defined as:

(18) $$\mathrm{CC}=\frac{cov(k,\hat{k})}{\sigma_{kk}\sigma_{\hat{k}\hat{k}}}$$

The CC between the estimated EOG artifact and the ground truth should be equal to 1 if an artifact removal approach successfully predicts the EOG artifact.

### 3.3. SAR

It is a metric that evaluates how much EOG artifact is removed from a raw EEG signal.

(19) $$\mathrm{SAR}=10log_{10}\frac{\sigma (k)}{\sigma (\hat{k}-k)}$$

### 3.4. MAE

We refer to the power spectrum of the contaminated and rectified EEG signals as $p_{s}\left(f\right)$ and $p_{\hat{q}}\left(f\right)$, respectively. The MAE among the spectrum of contaminated and rectified EEG signal is then determined as follows:

(20) $$\mathrm{MAE}=\frac{\sum_{f=a}^{b}\sum_{n=-\infty }\left|p_{s}\left(f\right)-p_{\hat{q}}\left(f\right)\right|}{a-b}$$

The indices of a particular band are its start and end frequencies, denoted by *a* and *b*. To examine the result of artifact suppression techniques on rectified EEG signal, the MAE is calculated for the α band. In the absence loss of EEG information due to an artifact elimination approach, the MAE value in a band should be near 0, indicating that the artifact removal approach is performing well.

## 4. Results

CiSSA-DWT was evaluated on synthetic EEG data and also real EEG signals to verify its efficacy in the following subsections.

### 4.1. Synthetic EEG Signal Results

**Figure 5:**
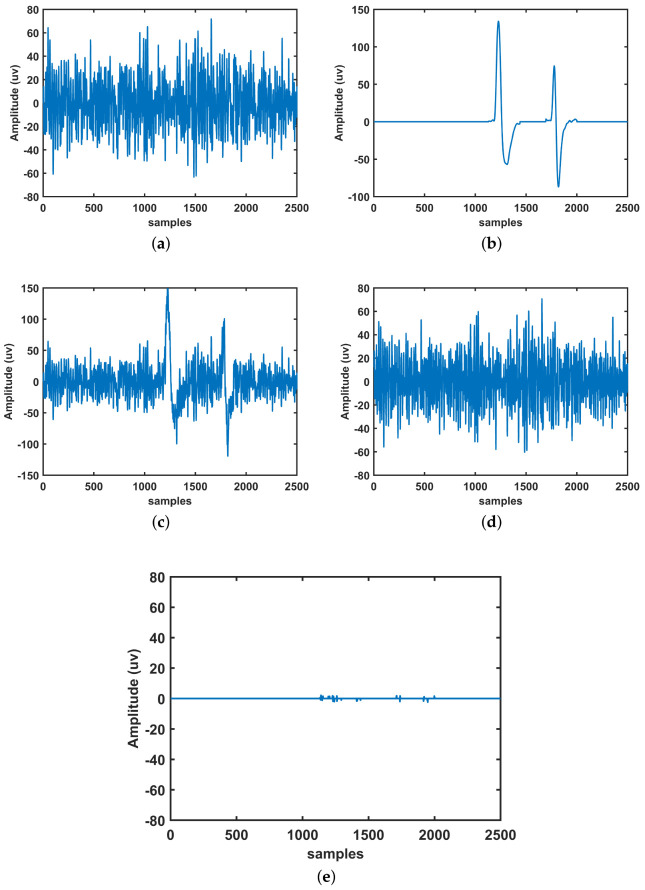
EOG removal in synthetic EEG data using proposed method. (**a**) Ground truth EEG signal. (**b**) Synthetic EOG artifact. (**c**) Contaminated EEG with EOG. (**d**) Filtered EEG using proposed method. (**e**) Variance signal by subtract filtered signal from original signal.

We use Equation (15) as the basis mixing model to assess the proposed performance of the model. The SNR value of the EEG signal is changed by the artifact mixing component *p*. We use EEG signals of 2500 samples each and a sampling frequency of 250 Hz to acquire data from the mendeley database [55]. As shown in Figure 5, the true EEG signal was combined with a synthetically created eye blink artifact (EOG) in order to create a contaminated EEG signal for the simulation experiment. At *p* = 1, Figure 5c depicts additive mixing of real EEG and EOG artifacts, resulting in a synthetically formed contaminated EEG signal. Moreover, we present EOG free EEG signal and comparison performance of the proposed method in Figure 5d and Figure 5e, respectively.

The EOG artifact is predicted in the proposed model from a single channel contaminated EEG signal using a novel approach (CiSSA-DWT) based on kurtosis and energy values. The initial phase of CiSSA in the proposed model is to create eigenvalues and vectors. The artifact component is then evaluated using diagonal averaging, which is followed by a grouping procedure. In general, the EOG component is found in the first one or two IMFs. Equations (8) and (10) were used to calculate the kurtosis and energy values of all IMFs in the proposed model. As an EOG artifact, the kurtosis and energy values of those IMFs exceed the threshold. For further decomposition, we add all of the EOG’s associated IMFs, which are then fed into a four-level DWT. The DWT produces both approximate and detailed coefficients. We removed the EOG artifact by eliminating the approximation coefficients, which are low frequency components. The inverse transformation of DWT of detailed coefficients was used to reconstruct the signal. By applying inverse CiSSA to all artifact-free components, the clean EEG signals finally reconstructed and Table 1 shows the mean values of RRMSE and CC.As a result, the good RRMSE and CC values were 12.1584 and 0.9935, respectively, attained at an artifact mixing constant of *p* = 1.5.

### 4.2. Real EEG Signal Results

**Figure 6:**
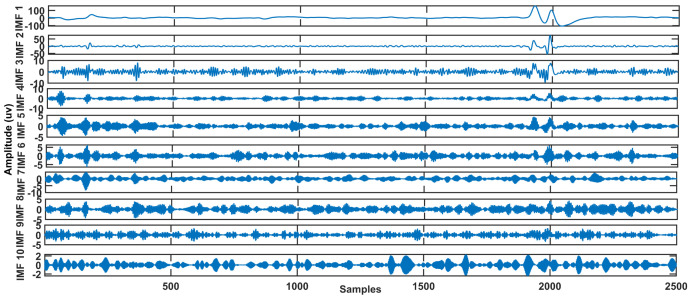
Decomposition of Raw EEG signal into IMFs using CiSSA.

**Figure 7:**
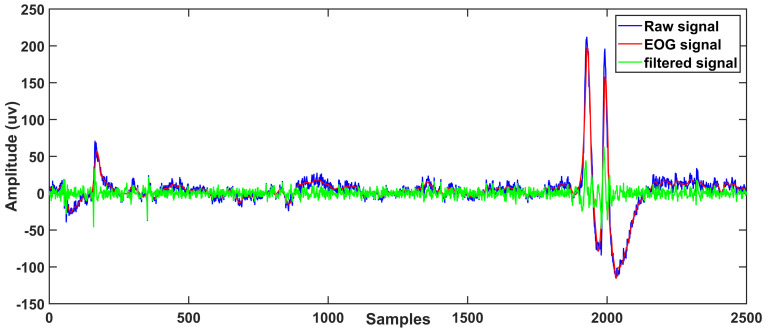
Clean EEG signal of extracted EOG artifact from raw EEG signal using proposed method.

The proposed approach was examined on real EEG data from dataset 2a of the BCI competition IV [51]. At a sampling frequency of 256 Hz, raw EEG data with EOG artifacts was acquired and recorded. The signal is separated into several segments, each with a length of 2500 samples. Our proposed method is applied to this data, and the findings of all segments of nine subjects records are averaged. SAR should be high for successful artifact removal algorithms. Table 2 and Table 3 show the processing results of proposed technique and CiSSA-DWT yields the lowest MAE and the highest SAR value. Figure 6 depicts the decomposition of raw EEG signal into IMFs and Figure 7 shows how the proposed technique reliably identifies the low frequency eye blink artifact-related reconstruction component and how DWT efficiently eliminates it while keeping low frequency content. As a result, the proposed CiSSA-DWT method achieved a good SAR value of 1.552 for subject 4 record 2. Similarly, the MAE value is 0.1034 for subject 2 record 1.

## 5. Discussion

**Figure 8:**
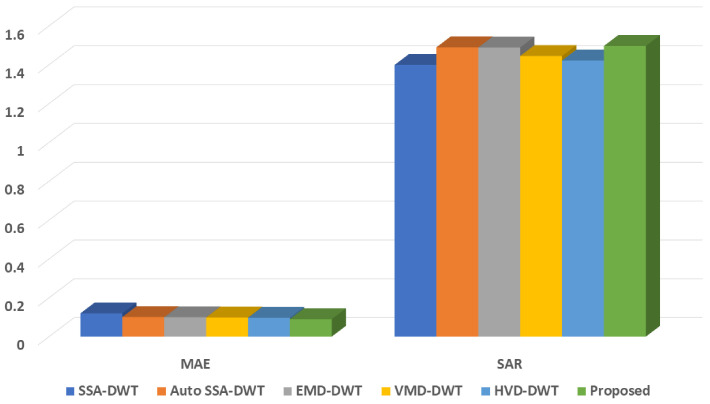
Comparison of MAE and SAR metrics with existing methods.

In this section, of the article, we described and compared the performance metrics of our proposed strategy with those of already existing methodologies and data-driven decomposition techniques. The performance of the recommended approach is compared to the performance of other current methods utilising the RRMSE and CC values acquired with reference to the ground truth eye blink artifact. This comparison is shown in Table 4. Under all circumstances, that is to say, *p* = 0.5, 1, 1.25, and 1.5, the recommended method produces mean RRMSE and CC values that are distinct from the RRMSE and CC values produced by the EEMD-ICA, the SSA-ICA, and the in [56] method. The estimated eye blink artifacts from either synthetic or actual EEG data cannot be retrieved using the previous method at either the beginning or the conclusion of the change-over points. Table 5 and Table 6 and a Figure 8 give comparable comparisons between the MAE and SAR values generated by the proposed method and the values derived from real EEG data, respectively. We were able to attain higher values for performance measures in comparison to other methods that were already in use [37,46].

On the other hand, we evaluated the data-driven decomposition methods, such as SSA-DWT, Auto-SSA-DWT, EMD-DWT, VMD-DWT, and HVD-DWT, based on the performance metrics presented in Table 5. These methods include SSA-DWT, Auto-SSA-DWT, EMD-DWT, VMD-DWT, and HVD-DWT. SSA is a non-parametric method that is used in the process of signal extraction based on singular value decomposition (SVD). The requirement to calculate the frequency of components after they have been removed is a drawback of the SSA method. IMFs are meant to be extracted using EMD all the way through the filtering and iteration steps. This results in the method having restrictions in several different areas, such as the frequency resolution and the effect of the sampling frequency. VMD separates the Fourier spectrum in order to retrieve the individual signal components in an organized fashion. In the same way that different components cannot be distinguished using the Fourier spectrum, VMD is unable to do so as well. As a result, this method is constrained in the same way as the Fourier spectrum is [60]. The HHT is a method for obtaining instantaneous frequency data and decomposing a signal into what are known as intrinsic mode functions (IMFs) and a trend. Additionally, the HHT can be used to determine a linear trend. It is designed to work effectively with data that is both nonstationary and nonlinear. Because mode mixing is one of the core problems with EMD, the theoretical foundation of HHT is completely empirical. They also outline some of the outstanding unresolved concerns with HHT, such as the unfavorable impacts of the EMD, issues with spline, the optimal choice of IMF, and uniqueness [38]. To extract individual components from nonstationary signals, Feldman invented a technique that he called the Hilbert Vibration Decomposition (HVD) [61]. The method is analogous to EMD in the sense that it continues to use an iterative recursive decomposition process on the residual signal from the step before it [60]. The Hilbert transform, often known as the HT, is an essential part of the HVD approach, as the name of this technique suggests. For the purpose of signal decomposition of EEG data, Sharma et al. [62,63] employed the sliding window transform (SWT) and sliding mode-SSA (SM-SSA) [64]. In [65], Multivariant variational mode decomposition (MVMD) and PCA were used for EOG artifact elimination and attained the CRC value of 0.632. The CiSSA methodology that has been developed is far more separable than other decomposition methods, which makes it much simpler to organize the elemental series in order to generate the various signals. CiSSA is a more automated process, and the components that it creates are more distinct. We conclude by making a few observations that should be taken into account in future work:

- The machine learning algorithms are not entirely explored in this research domain. Therefore, the scope of machine learning and deep learning approaches in ocular artifact removal from the EEG signal should be expanded.
- Hybrid approaches should be enhanced with some additional processing stages to eliminate all the types of artifacts present in the EEG signal, as hybrid yields promising results and is growing in this area of research.
- Advancements should be made to prior algorithms to develop a uniform criterion for the validation of EEG signals acquired in clinical studies.

### Experiment on Parameters Tuning

**Figure 9:**
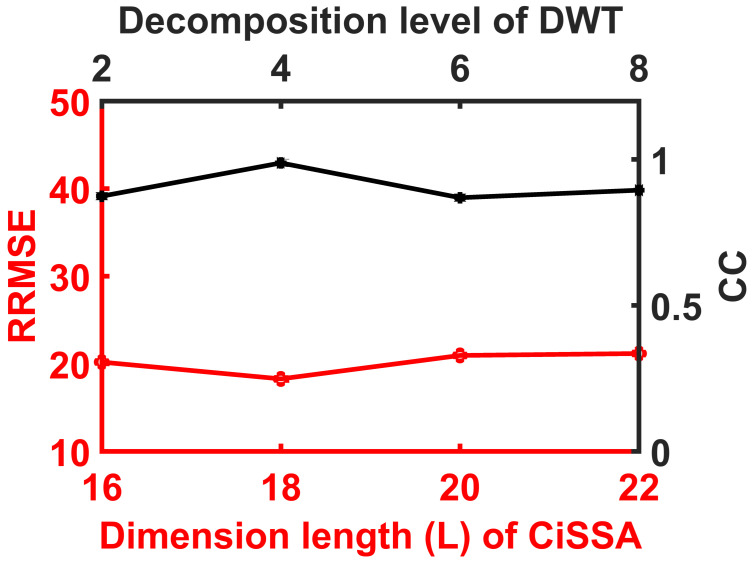
Plot shows variance of RRMSE and CC values of CiSSA-DWT tuning parameters.

**Figure 10:**
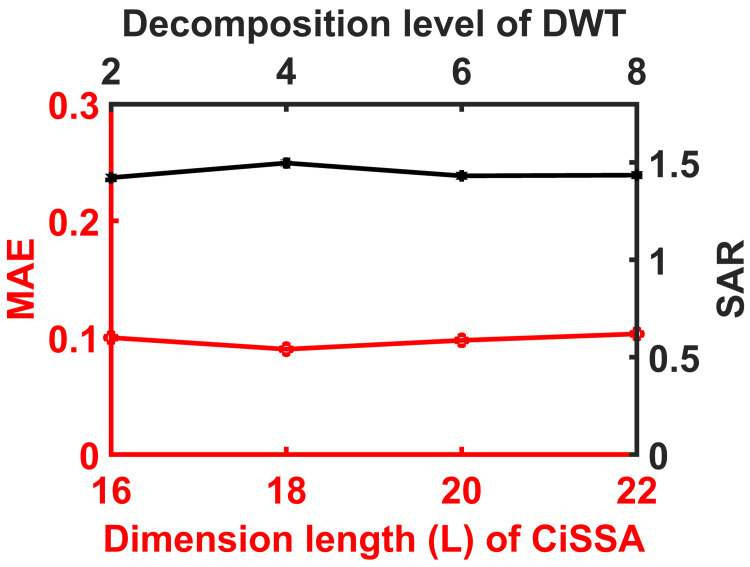
Plot shows variance of MAE and SAR values of CiSSA-DWT tuning parameters.

CiSSA and DWT tuning parameters, such as embedding dimension (L) and level of decomposition, are critical. Figure 9 and Figure 10 show the results of testing the recommended approach for various values of this parameter. As a result, L = 18 and four-level DWT decomposition yielded a superior result, and these parameters were used throughout the study. When compared to DWT tuning parameter CiSSA is important because the parameters variance is very small on synthetic and real EEG data performance metrics of DWT as shown in Figure 9 and Figure 10.

## 6. Conclusions

A novel CiSSA-DWT was developed for the reliable elimination of the EOG artifact for single channel EEG signals. To estimate EOG artifacts, two simple aspects of the EEG signal were used, and the CiSSA approach was used to eliminate EEG remnants from the calculated EOG artifact. The suggested approach eliminates the EOG artifact without affecting the original EEG components, according to the results. Because two features, kurtosis and energy, are effective at distinguishing the EEG and EOG components, the suggested technique was able to eliminate the EOG artifact from the EEG signals by fine-tuning its parameters. A comparison of the proposed technique to current and existing EOG artifact removal methods shows that it is better in terms of precise filtering of EOG artifacts. The proposed approach (CiSSA-DWT) can be used for EEG preprocessing in applications that just use a few or single frontal EEG channels.

## Figures and Tables

**Table 1:** Mean values of RRMSE and CC with different *p* values.

Artifact Mixing Constant (*p*)	RRMSE	CC
***p*** ** = 0.5**	29.8156	0.9686
***p*** ** = 1**	18.2743	0.9883
***p*** ** = 1.25**	15.1146	0.9892
***p*** ** = 1.5**	12.1584	0.9935

**Table 2:** MAE values of real EEG signals.

Subjects	Records								
	R-1	R-2	R-3	R-4	R-5	R-6	R-7	R-8	Average
**Subject 1**	0.0901	0.0855	0.0824	0.0908	0.0800	0.0787	0.0970	0.0852	**0.0862**
**Subject 2**	0.1034	0.0917	0.0996	0.0899	0.0777	0.0889	0.0794	0.0901	**0.0900**
**Subject 3**	0.0806	0.0881	0.0999	0.0834	0.0801	0.088	0.0911	0.0887	**0.0874**
**Subject 4**	0.0855	0.0712	0.0767	0.0786	0.0842	0.0789	0.0799	0.0863	**0.0801**
**Subject 5**	0.0933	0.0899	0.0779	0.1000	0.0810	0.0872	0.0909	0.0869	**0.0883**
**Subject 6**	0.0900	0.0891	0.0893	0.0781	0.0910	0.0842	0.0999	0.0794	**0.0876**
**Subject 7**	0.0934	0.0812	0.0895	0.0887	0.0745	0.0877	0.0774	0.0885	**0.0851**
**Subject 8**	0.0843	0.0784	0.0796	0.0896	0.0892	0.0888	0.0909	0.0772	**0.0847**
**Subject 9**	0.0981	0.0912	0.0886	0.0987	0.0871	0.0788	0.0804	0.0900	**0.0891**

**Table 3:** SAR values of real EEG signals.

Subjects	Records								
	R-1	R-2	R-3	R-4	R-5	R-6	R-7	R-8	Average
**Subject 1**	1.399	1.497	1.410	1.320	1.278	1.271	1.355	1.389	**1.362**
**Subject 2**	1.394	1.454	1.352	1.313	1.324	1.356	1.311	1.291	**1.349**
**Subject 3**	1.237	1.401	1.131	1.401	1.410	1.322	1.404	1.314	**1.328**
**Subject 4**	1.394	1.552	1.445	1.407	1.412	1.453	1.476	1.481	**1.453**
**Subject 5**	1.121	1.259	1.261	1.211	1.228	1.265	1.110	1.121	**1.197**
**Subject 6**	1.330	1.260	1.347	1.424	1.385	1.410	1.300	1.427	**1.360**
**Subject 7**	1.402	1.120	1.220	1.400	1.444	1.268	1.405	1.200	**1.307**
**Subject 8**	1.279	1.335	1.290	1.369	1.284	1.318	1.264	1.287	**1.303**
**Subject 9**	1.333	1.399	1.305	1.461	1.290	1.375	1.463	1.412	**1.380**

**Table 4:** Comparison of proposed method RRMSE and CC values with existing methods.

Authors	Methodology	*p* = 0.5		*p* = 1		*p* = 1.25		*p* = 1.5	
		RRMSE	CC	RRMSE	CC	RRMSE	CC	RRMSE	CC
B. Mijovic et al. [56]	EEMD-ICA	67.8152	0.8297	37.485	0.9358	31.212	0.9529	26.863	0.964
Giuseppina Inuso et al. [57]	SSA-ICA	61.1533	0.8522	32.585	0.9499	27.21	0.964	22.78	0.9746
Zhang, S. et al. [58]	Infomax and FastICA	42.4855	0.9159	20.168	0.9754	18.632	0.9794	16.283	0.9819
Gajbhiye, P. et al. [59]	FBSE-EWT	31.3118	0.9513	20.203	0.9801	17.067	0.9858	14.709	0.9895
**Proposed**	**CiSSA-DWT**	**29.8156**	**0.9686**	**18.274**	**0.9883**	**15.115**	**0.9892**	**12.158**	**0.9935**

**Table 5:** Comparison of other data-driven decomposition methods with CiSSA-DWT method.

Method	MAE	SAR	RRMSE	CC
SSA-DWT	0.1201	1.399	32.9877	0.954
Auto SSA-DWT	0.1010	1.490	30.6571	0.962
EMD-DWT	0.1000	1.489	30.7426	0.958
VMD-DWT	0.0988	1.445	24.2870	0.964
HVD-DWT	0.0972	1.422	20.2033	0.975
**CISSA-DWT**	**0.0901**	**1.497**	**18.2743**	**0.988**

**Table 6:** Comparison of proposed method MAE and SAR values with existing methods.

Authors	Methodology	MAE	SAR
Giuseppina Inuso et al. [57]	W-ICA	0.092	1.812
A.K. Maddirala et.al [46]	SSA-ANC	0.1001	1.52
B. Mijovic et al. [56]	EEMD-ICA	0.1009	1.752
Maddirala et al. [47]	SSA-ICA	0.1007	1.589
Zhang, S. et al. [58]	Infomax and FastICA	0.736	1.7789
Gajbhiye, P. et al. [59]	FBSE-EWT	0.3428	1.634
**Proposed**	**CiSSA-DWT**	**0.0801**	**1.4525**

## Data Availability

The publicly available BCI Competition IV Data set 2a was used in this work [51].
